# Anastomotic Leakage in Emergency Colorectal Surgery: Clinical Features and Associated Variables

**DOI:** 10.7759/cureus.106070

**Published:** 2026-03-29

**Authors:** Daniel Iván Astorga Guardado, Ana J Iberri Jaime, Billy Jiménez Bobadilla

**Affiliations:** 1 Colorectal Surgery, General Hospital of Mexico Dr. Eduardo Liceaga, Mexico City, MEX; 2 Coloproctology, General Hospital of Mexico Dr. Eduardo Liceaga, Mexico City, MEX

**Keywords:** anastomotic leakage, colorectal anastomotic leak, colorectal surgeon, general emergency surgery, risk factors

## Abstract

Background

Anastomotic leakage (AL) is one of the most serious complications in colorectal surgery and is associated with increased morbidity, prolonged hospital stay, and mortality. Emergency colorectal procedures carry a higher risk of AL due to unfavorable clinical conditions and patient factors.

Methods

A retrospective cohort study was conducted at the General Hospital of Mexico “Dr. Eduardo Liceaga,” a tertiary referral center in Mexico City, including patients who underwent emergency colorectal resection with primary anastomosis between January 2017 and June 2024. The primary outcome was AL. Clinical, surgical, and laboratory variables were analyzed. Continuous variables were compared using the Mann-Whitney U test, and categorical variables using Fisher’s exact test.

Results

A total of 52 patients were included. AL occurred in 11.5% of patients (n = 6). Patients with AL had a significantly longer hospital stay compared to those without AL (17.5 vs 8.5 days, p = 0.037). Lower preoperative albumin levels were significantly associated with AL (3.63 vs 4.01 g/dL, p = 0.046). C-reactive protein showed a trend toward significance (p = 0.055), while other variables were not significantly associated with AL. A higher proportion of AL was observed in patients with colorectal cancer (20.0%, 4/20) compared to those with diverticular disease (6.5%, 2/31). All cases of AL required surgical reintervention. Postoperative complications occurred in 46.1% of patients (n = 24), and mortality was observed in 1.9% (n = 1), occurring in a patient with AL.

Conclusions

AL remains a significant complication in emergency colorectal surgery. Lower preoperative albumin levels were associated with an increased risk of AL, and patients with AL experienced worse postoperative outcomes. Further studies are needed to better define risk factors and optimize management strategies.

## Introduction

Colorectal surgery is associated with clinically relevant morbidity and mortality, with reported complication rates ranging from 20% to 35% and a 30-day mortality between 2% and 9% [1-4]. Among these complications, anastomotic leakage (AL) represents one of the most severe adverse events, with a reported incidence ranging from 3% to 20%, depending on the population and clinical setting, and is associated with significantly increased postoperative morbidity and mortality, particularly when accompanied by peritonitis or sepsis [3,5].

The definition of AL has evolved over time. Currently, it is widely accepted as a defect of the intestinal wall at the anastomotic site that results in communication between the intra- and extraluminal compartments, as established by the International Study Group of Rectal Cancer [5]. This standardization has allowed more consistent reporting and comparison across studies.

The etiology of AL is multifactorial and involves patient-related factors, clinical context, and technical aspects of the surgical procedure. Risk factors for AL include male sex, smoking, comorbidities, intraoperative blood loss, prolonged operative time, and exposure to preoperative chemotherapy or radiotherapy [6-10]. In addition, a high American Society of Anesthesiologists (ASA) score and emergency surgical indication have been consistently associated with an increased risk of clinically significant AL [9].

Despite extensive research, most available evidence derives from elective colorectal surgery populations. Predictive models and scoring systems have been developed; however, their clinical applicability remains limited due to heterogeneity in variables and validation in small or highly selected cohorts [6,7].

In scenarios involving benign disease, such as complicated acute diverticulitis, primary anastomosis has been increasingly supported as a safe alternative to Hartmann’s procedure in selected patients. Recent evidence, including large retrospective cohort analyses using propensity score matching, has demonstrated comparable or improved outcomes with primary anastomosis, even in advanced disease stages such as Hinchey III or IV [11].

In emergency colorectal surgery, patients are exposed to additional risk factors such as hemodynamic instability, contamination, and suboptimal physiological conditions, which may further compromise anastomotic healing. However, data focusing specifically on AL in emergency settings remains limited, particularly in Latin American populations.

Understanding the factors associated with AL in emergency colorectal surgery is essential to improve surgical decision-making and optimize patient outcomes.

The aim of this study was to identify factors associated with AL in patients undergoing emergency colorectal surgery in a tertiary referral center.

## Materials and methods

Study design and setting

A retrospective cohort study was conducted at the General Hospital of Mexico “Dr. Eduardo Liceaga,” a tertiary referral center in Mexico City. Medical records of patients who underwent emergency colorectal surgery between January 2017 and June 2024 were reviewed.

Population and eligibility criteria

Adult patients (≥18 years) who underwent emergency colorectal resection with primary anastomosis were included.

Exclusion criteria were patients without primary anastomosis, incomplete medical records, and patients lost to postoperative follow-up.

Outcome definition

The primary outcome was AL, defined according to the International Study Group of Rectal Cancer as a defect of the intestinal wall at the anastomotic site resulting in communication between the intra- and extraluminal compartments.

AL was identified based on clinical, radiological, or intraoperative findings, including signs of peritonitis, feculent or purulent drainage through surgical drains or wounds, radiological evidence of leakage on imaging studies, and confirmation during reoperation

Variables

The following variables were collected: demographic variables (age and sex), comorbidities (hypertension, diabetes mellitus, chronic kidney disease, and cardiovascular disease), clinical variables (smoking status, alcohol use, and ASA classification), laboratory variables (preoperative albumin, C-reactive protein (CRP), leukocyte count, and hemoglobin levels), surgical variables (surgical approach (open vs. laparoscopic), type of surgeon (colorectal vs. general), operative time, previous abdominal surgery, and presence of protective stoma), and postoperative variables (length of hospital stay, need for reintervention, and postoperative complications classified according to the Clavien-Dindo classification).

Statistical analysis

Continuous variables were assessed for normality using the Shapiro-Wilk test. As most variables did not follow a normal distribution, they were reported as median and interquartile range (IQR) and compared using the Mann-Whitney U test.

Categorical variables were expressed as frequencies and percentages and compared using Fisher’s exact test. A p-value < 0.05 was considered statistically significant. All statistical analyses were performed using IBM SPSS Statistics version 30.0 (IBM Corp., Armonk, NY, USA).

Ethical considerations

This study was approved by the institutional ethics committee of the General Hospital of Mexico. Given its retrospective nature, informed consent was waived. Patient confidentiality was maintained in accordance with the Declaration of Helsinki.

## Results

A total of 130 medical records were reviewed, of which 52 patients met the inclusion criteria and were included in the analysis.

The median age of the cohort was 50 years (interquartile range (IQR): 41-61), and 63.5% (n = 33) were male. AL occurred in 11.5% of patients (n = 6). All cases of AL required surgical reintervention and were managed with stoma creation.

Comparison between patients with and without AL

Continuous variables are summarized in Table 1. Patients who developed AL had a higher median age compared to those without AL (62.5 vs. 48.0 years), although this difference did not reach statistical significance (p = 0.080). Median operative time was not significantly different between groups (230 vs. 275 minutes, p = 0.576). However, patients with AL had a significantly longer hospital stay compared to those without AL (17.5 vs. 8.5 days, p = 0.037).

Laboratory variables

As shown in Table 1, patients with AL had significantly lower preoperative albumin levels compared to those without AL (3.63 vs. 4.01 g/dL, p = 0.046). C-reactive protein (CRP) levels showed a trend toward statistical significance (0.93 vs. 7.11 mg/L, p = 0.055). Leukocyte count and hemoglobin levels were not significantly different between groups (p = 0.819 and p = 0.742, respectively).

Categorical variables

Categorical variables are presented in Table 2. No statistically significant associations were identified between AL and categorical variables using Fisher’s exact test. However, a higher proportion of AL was observed in patients with a smoking history (83.3% vs. 39.1%, p = 0.076) and in those with chronic kidney disease (33.3% vs. 6.5%, p = 0.096), although these findings did not reach statistical significance.

Anastomotic leakage according to diagnosis

AL rates according to diagnosis are shown in Table 3. Patients with colorectal cancer had an AL rate of 20.0% (4/20), while those with diverticular disease had an AL rate of 6.5% (2/31). Although these differences were not statistically significant, this descriptive analysis provides additional clinical context.

Postoperative outcomes

Postoperative complications occurred in 46.1% of patients (n = 24). The distribution of complications according to the Clavien-Dindo classification is presented in Table 4. Most patients had no complications (grade 0), while complications were distributed as follows: grade I in 28.8% (n = 15), grade II in 7.7% (n = 4), grade IIIb in 11.5% (n = 6), and grade IV in 9.6% (n = 5).

All cases of AL required surgical reintervention and were therefore classified as Clavien-Dindo grade IIIb. Some patients additionally developed severe complications requiring intensive care support, corresponding to grade IV.

A total of 13.5% of patients (n = 7) required surgical reintervention. Six of these cases corresponded to AL, while one additional patient without AL required reintervention for another postoperative complication.

Mortality was observed in 1.9% of patients (n = 1), and this case occurred in a patient with AL.

Table 1 shows the comparison between patients with and without AL.

**Table 1:** Comparison between patients with and without anastomotic leakage Continuous variables are presented as median (interquartile range) and compared using the Mann–Whitney U test. Categorical variables were compared using Fisher’s exact test. AL: anastomotic leakage, CRP: C-reactive protein

Variable	No AL (n = 46)	AL (n = 6)	p-value	U statistic
Age (years)	48 (40–60)	62.5 (60–70.7)	0.080	199.5
Operative time (min)	275 (255–320)	230 (187–512)	0.576	118
Hospital stay (days)	8.5 (6–11.5)	17.5 (12.7–20.7)	0.037	211
Albumin (g/dL)	4.01 (3.61–4.19)	3.63 (2.75–3.85)	0.046	68
CRP (mg/L)	7.11 (1.5–15.9)	0.93 (0.43–5.53)	0.055	70.5
Leukocytes (×10³/µL)	11.75 (7.07–15.6)	9.7 (8.85–13.4)	0.819	129.5
Hemoglobin (g/dL)	14.35 (13.5–15.48)	14.65 (11.5–15.14)	0.742	126

Table 2 shows the categorical variables associated with AL.

**Table 2:** Categorical variables associated with anastomotic leakage Categorical variables are presented as frequencies and percentages. Comparisons were performed using Fisher’s exact test. AL: anastomotic leakage

Variable	No AL (n = 46)	AL (n = 6)	p-value
Male sex	29 (63.0%)	4 (66.7%)	1.000
Hypertension	18 (39.1%)	2 (33.3%)	1.000
Diabetes mellitus	9 (19.6%)	1 (16.7%)	1.000
Chronic kidney disease	3 (6.5%)	2 (33.3%)	0.096
Smoking	18 (39.1%)	5 (83.3%)	0.076
Alcohol use	15 (32.6%)	3 (50.0%)	0.392
Previous abdominal surgery	28 (60.9%)	3 (50.0%)	0.675
Protective stoma	6 (13.0%)	1 (16.7%)	1.000
Laparoscopic approach	10 (21.7%)	1 (16.7%)	1.000
Colorectal surgeon	30 (65.2%)	3 (50.0%)	0.648

Table 3 shows the AL according to diagnosis.

**Table 3:** Anastomotic leakage according to diagnosis Anastomotic leakage (AL) rates are presented according to underlying diagnosis. No statistically significant differences were observed.

Diagnosis	Total (n)	AL (n)	AL (%)
Colorectal cancer	20	4	20.0%
Diverticular disease	31	2	6.5%

Table 4 shows the postoperative complications according to the Clavien-Dindo classification.

**Table 4:** Postoperative complications according to the Clavien–Dindo classification Postoperative complications were classified according to the Clavien–Dindo classification system. Grade IIIb includes patients requiring surgical reintervention.

Grade	n	%
0 (no complications)	28	53.8%
I	15	28.8%
II	4	7.7%
IIIb	6	11.5%
IV	5	9.6%

## Discussion

AL remains one of the most feared complications in colorectal surgery due to its association with increased morbidity, prolonged hospitalization, and mortality [5,9]. In this study, the incidence of AL was 11.5%, which is within the upper range reported in the literature, particularly for emergency colorectal surgery, a setting known to carry higher risk compared to elective procedures [3,5].

One of the most relevant findings of this study was the significant association between lower preoperative albumin levels and the development of AL. Hypoalbuminemia is a well-recognized marker of poor nutritional status and systemic inflammation, both of which impair wound healing and anastomotic integrity [7,10]. This finding is consistent with previous studies that have identified albumin as an important predictor of postoperative complications, including AL [7,10]. From a clinical perspective, this reinforces the importance of preoperative optimization whenever feasible, even in urgent scenarios.

Additionally, patients who developed AL had a significantly longer hospital stay, reflecting the clinical impact and resource utilization associated with this complication. This finding has been consistently reported in prior studies and reinforces the burden associated with AL [5].

Although not statistically significant, several variables showed a trend toward association with AL, particularly smoking and chronic kidney disease. Smoking has been linked to impaired tissue oxygenation and microvascular dysfunction, while chronic kidney disease is associated with systemic inflammation and immune dysregulation, all of which may contribute to impaired healing [9,10]. The lack of statistical significance in these variables is likely related to the limited sample size and the low number of events.

Interestingly, no significant differences were observed in operative time, surgical approach, or type of surgeon. While these factors have been associated with AL in other studies, their impact may be less pronounced in emergency settings, where patient-related and physiological factors play a more dominant role [8,9].

Regarding laboratory parameters, CRP showed a trend toward significance but did not reach statistical significance. This may be explained by variability in the timing of measurement and the retrospective nature of the study. Leukocyte count and hemoglobin levels were not associated with AL, suggesting that these markers may have limited predictive value in this specific clinical context.

When stratified by diagnosis, a higher rate of AL was observed in patients with colorectal cancer compared to those with diverticular disease (20.0% vs. 6.5%). Although this difference was not statistically significant, it may reflect the more complex nature of oncologic resections and the potential impact of tumor-related factors. These findings are consistent with previous reports suggesting increased risk in cancer-related resections [9].

In terms of postoperative outcomes, all cases of AL required surgical reintervention and were therefore classified as Clavien-Dindo grade IIIb. Furthermore, some patients developed severe complications requiring intensive care, corresponding to grade IV. Importantly, the only mortality observed in this cohort occurred in a patient with AL, highlighting the clinical severity of this complication and its direct impact on patient outcomes [5].

This study has several limitations. First, its retrospective design introduces the possibility of selection and information bias. Second, the relatively small sample size and low number of AL events limit the statistical power to detect significant associations and precluded the use of multivariable analysis due to the risk of model overfitting. Third, although several relevant variables were included, other potentially important factors may not have been captured or consistently recorded.

Despite these limitations, this study provides valuable insight into factors associated with AL in emergency colorectal surgery, a setting that remains underrepresented in the literature, particularly in Latin American populations. The identification of hypoalbuminemia as a significant factor and the observed trends in smoking and chronic kidney disease may help guide clinical decision-making and risk stratification.

## Conclusions

AL remains a significant complication in emergency colorectal surgery, with a considerable impact on postoperative outcomes. In this study, lower preoperative albumin levels were significantly associated with the development of AL, highlighting the importance of nutritional and inflammatory status in anastomotic healing.

Patients who developed AL experienced longer hospital stays and higher postoperative morbidity, and the only mortality observed in this cohort occurred in a patient with AL, underscoring the clinical severity of this complication.

Although other variables such as smoking and chronic kidney disease showed a trend toward association with AL, these findings did not reach statistical significance, likely due to the limited sample size.

These results suggest that preoperative risk assessment, particularly focusing on modifiable factors such as nutritional status, may help improve outcomes in emergency colorectal surgery. Further prospective studies with larger sample sizes are needed to better define risk factors and optimize management strategies.
